# Long-Term Outcomes of Digital Nerve Repair Accompanied by Digital Artery Injury in Flexor Zone 2

**DOI:** 10.1055/s-0039-3400229

**Published:** 2019-12-02

**Authors:** Gokce Yildiran, Mustafa Sutcu, Osman Akdag, Zekeriya Tosun

**Affiliations:** 1 Division of Hand Surgery, Department of Plastic, Reconstructive and Aesthetic Surgery, Selcuk University Medical Faculty, Konya, Turkey

**Keywords:** artery repair, digital nerve, finger, nerve healing

## Abstract

**Objectives** Better healing results of any tissue or area is closely linked with a well-blood supply in reconstructive surgery. Peripheric nerve healing is closely related to blood supply as well. We aimed to assess whether there was any difference between digital nerve healing with and without extrinsic blood supply.

**Methods** We assessed 48 patients with unilateral digital nerve injury at zone 2. Twenty-four of them had unrepairable arterial injury and other 24 had no arterial injury. The 24 patients in the “unrepaired artery group” (UA) and 24 patients in the “intact artery group” (IA) were compared.

**Results** Mean follow-up time was 17.7 months. The mean two-point discrimination (2PD) was 5.29 mm in IA group and 5.37 mm in UA group. One neuroma in IA group and two neuromas in UA group were determined. We found no statistically significant difference between these groups in terms of neuroma, 2PD, and cold intolerance. The results of British Medical Research Council sensory recovery clinical scale were comparable for these two groups.

**Conclusion** Digital nerve healing is related to numerous factors. We hypothesized that blood flow may be one of these factors; however, at this zone digital artery repair is not the foremost determinant for digital nerve healing. Further researches should be done for upper injury levels. Despite this result, we argue not to leave the digital artery without repairment and we propose to repair both artery and nerve to achieve the normal anatomical integrity and to warrant finger blood flow in possible future injuries.

Digital nerve injuries are common and often require epineural repair. 1 2 Age, trauma type, and smoking habit affect digital nerve healing; however, no consensus exists regarding the outcomes of epineural nerve repair for digital nerve injuries. 3 4

Blood flow to the digital nerves is through the digital artery. 5 Nerve repair is often prioritized as long as the finger is not devascularized, and arterial repair may prolong the duration of surgery. However, there is little data in the literature pertaining to the effects of restored arteries on nerves. 6 We hypothesized that “better the blood supply, better the healing” for digital nerves. Therefore, we aimed to elucidate the role of the digital artery on digital nerve healing and whether a functional digital artery improves the clinical outcomes of pulp sensitivity.

## Materials and Methods

Patients who underwent digital nerve repair from January 2012 to April 2015 were retrospectively evaluated. Patients who presented with no notable arterial disease and whose epineural nerve repairs were performed by the same surgeon primarily using 9/0 polyamide sutures (three sutures) under a microscope within 12-hour were included in the study. Patients with a single cut injury at the flexor zone 2 level were included, and no tissue adhesive or tubulization was used in any patient. A cast was applied in all patients after surgery.

The patients were divided in to two study groups ( *n* = 24 each): patients who underwent digital nerve repair for both digital nerve and artery injuries although whose digital arteries were unrepairable for any reason were classified into “unrepaired artery” (UA) group and patients who underwent digital nerve repair for digital nerve injuries only, as selected by a surgeon via systemic random sampling were classified into “intact artery” (IA) group.

Age, sex, smoking status, and follow-up duration were recorded.

Patients with crush injuries, artery-only injuries, both nerve and artery repairs, nerve grafts, bilateral digital nerve injuries, replantations, repairs by another surgeon, accompanying tendon injuries, tendon lacerations, and bone or joint pathologies were excluded from the study.

Patients in the UA and IA groups were compared, and a minimum of 1-year data were evaluated. Data on the mean age, sex, smoking status, and follow-up durations were derived using clinical archives. Two-point discrimination (2PD) was measured using caliper, and sensory recovery was evaluated using the British Medical Research Council (MRC) clinical scale. 7 Neuroma was evaluated by physical examination and Tinel's sign. 8 In addition, patients were questioned with Cold Intolerance Severity Score (CISS) regarding cold intolerance. 9 The patency of the digital artery was evaluated using Doppler ultrasonography. Postoperative finger immobilization was applied for 1 week. The two groups were compared according to their 2PD results and neuroma formation and evaluated using *t* -test and chi-squared test.

## Results

The mean age was 45.5 (17–84) years in the IA group and 49.8 (17–84) years in the UA group. Both groups comprised 17 females and 7 males. In total, 14 patients in the IA group and 17 patients in the UA group were smokers. The mean follow-up duration was 17.7 months (12–50 months) in the IA group and 21.4 months (13–43 months) in UA group.

One neuroma was detected in the IA group and two were detected in the UA group. The mean 2PD was 5.29 (4–8) mm in the IA group and 5.37 (4–10) mm in the UA group. Three cold intolerance (>50 points of CISS) was detected in the IA group and five (>50 points of CISS) were detected in the UA group ( Table 1 ). No statistically significant differences found between the two groups in terms of neuroma formation ( *p* > 0.05), 2PD ( *p* > 0.05) and cold intolerance ( *p* > 0.05). According to the MRC clinical scale for grading sensory recovery; one grade S3, two grade S4, twenty-one grade S5 were detected in the IA group and two grade S3, three grade S4, nineteen grade S5 were detected in the UA group. The results were comparable for these two groups.

**Table 1:** Results for IA group and UA group

	IA group	UA group
*Mean age*	45.5	49.8
*Smokers (n)*	14	17
*Mean follow-up (months)*	17.7	21.4
*Neuroma (n)*	1	2
*Mean 2PD (mm)*	5.29	5.37
*Cold intolerance (n >* *50 points of CISS)*	3	5

Abbreviations: 2PD, two-point discrimination; CISS, Cold Intolerance Severity Score; IA, intact artery; UA, unrepaired artery.

## Discussion

Predicting the repair outcomes of peripheral nerve injuries is challenging. Approaches to improve healing remain ambiguous despite the knowledge that many factors, such as smoking habit, vitamin use, age, trauma, concomitant illnesses, and repair type, can either positively or negatively affect nerve recovery. 10 However, the healing of a tissue or an area is associated with a good blood supply in reconstructive surgery, particularly for soft tissues, bones, flaps, and tendons. 11 12

Vascularized nerve grafts were first presented in the literature in 1976, and the theory “well-nourished, well-heals” was proposed. 13

In the literature, significantly better results were reported in cases that underwent early repairs and required a short duration of denervation. 10 In another study, Terzis and Kostopoulos have reported good and excellent results in the upper extremity using vascularized nerve grafts in scar tissue previously treated with nerve grafts. 14 Similarly, the disruption of nerve blood supply has been shown to cause central necrosis in the nerve and the failure of nerve regeneration. 15

The blood supply to peripheral nerves is provided by an extrinsic as well as an extensive intrinsic network. This vascular network is crucial owing to the high metabolic demands of nerve tissues. 16

The decreased blood perfusion of the vasa nervorum in peripheral nerves impairs nerve healing by creating a poor environment for reinnervation. 17

We aimed to assess whether there was any difference between digital nerve healing with and without an extrinsic blood supply.

In the present study, one of the groups presented with no arterial injury, and all patients in this group underwent nerve repair for nerve injury only. In the other group, all patients presented with both nerve and arterial injury. All patients in this group underwent nerve repair, but arterial repair was not possible.

In both the groups, none of the patients presented with any repaired digital artery injury. Patients who had undergone both arterial and nerve repairs were not included in the study because the repaired arteries would have demonstrated a continued patency and would have biased the study.

In our study, all nerve repairs were performed within the first 12 hours after injury because Wallerian degeneration starts within 12 to 48 hours in an injured nerve.

Notably, the type of injury is important because the extent of damage to blood supply increases as more scars develop with an increasing number of dissections around a tissue, thereby impairing nerve healing. Thus, only isolated and single incisional injuries at the flexor zone 2 were included in this study.

The mean 2PD was 5.29 mm in the IA group and 5.37 mm in the UA group. According to the MacKinnon 2PD classification, ≤ 6mm is the “excellent” quality of sensation. 18 Achieving these excellent outcomes even in cases in which the artery had not been repaired demonstrated that several other factors are involved in nerve healing rather than the blood supply alone. Conversely, unilateral injuries in this zone may not impair the nerve blood supply as much since this would create scar tissue owing to the other digital bundle.

In the present study, only the extrinsic system was evaluated in the UA group. When the digital nerve is repaired, even if the digital artery cannot be repaired, the intrinsic blood supply system of the nerve may be re-established because the intrinsic system is extensive and linked with the mesoneurium, endoneurium, and perineurium. 19

However, we do not believe that it is acceptable to not repair the digital artery. In our opinion, every physician dealing with hand surgery should be aware that injury to the digital bundle may recur in the same patient. Although repairing the digital artery does not improve digital nerve healing, we argue that the digital artery must be repaired to maintain normal anatomic structure and to maximize finger blood flow in case of possible future injuries.

The present study has some limitations. This was a retrospective study, and we believe that clinical nerve healing studies are challenging for clinicians to design because of the different types of injuries and different healing capacities among individuals. Furthermore, this study only assessed digital nerve healing at the flexor zone 2 among single cut injuries. The effect of repair of extrinsic arteries in higher zones should be further investigated for nerve regeneration.

In conclusion, maintaining blood supply by performing arterial repair may not be the priority for the effective recovery of the digital nerve. The present study demonstrates that whether the digital artery is intact or not cannot be a marker for predicting nerve healing at the flexor zone 2.
